# Supplementary material for: Sensitization of Patient-Derived Colorectal Cancer Organoids to Photon and Proton Radiation by Targeting DNA Damage Response Mechanisms
Source: Cancers (Basel). 2022 Oct 11;14(20):4984. doi: 10.3390/cancers14204984 (PMC9599341; doi:10.3390/cancers14204984)
Supplement: Supplementary file 1 [file cancers-14-04984-s001.zip › cancers-1899420-supplementary.pdf]

cancers

MDPI

Supplementals

Sensitization of Patient-Derived Colorectal Cancer Organoids to Photon and Proton Radiation by Targeting DNA Damage Response Mechanisms

Kristin Pape [1,2,†], Anna J. Lößner [1,2,†], Doreen William [3,4,5], Tabea Czempiel [3,4,5], Elke Beyreuther [6,7], Anna Klimova [2], Claudia Lehmann [1,2], Tim Schmäche [1,2], Sebastian R. Merker [1,2], Max Naumann [4,6], Anne-Marlen Ada [1,2], Franziska Baenke [1,2], Therese Seidlitz [1,2], Rebecca Bütof [2,6,8,9], Antje Dietrich [4,6], Mechthild Krause [2,4,6,8,9], Jürgen Weitz [1,2], Barbara Klink [2,3,4,5,11], Cläre von Neubeck [4,6,10] and Daniel E. Stange [1,2,*]

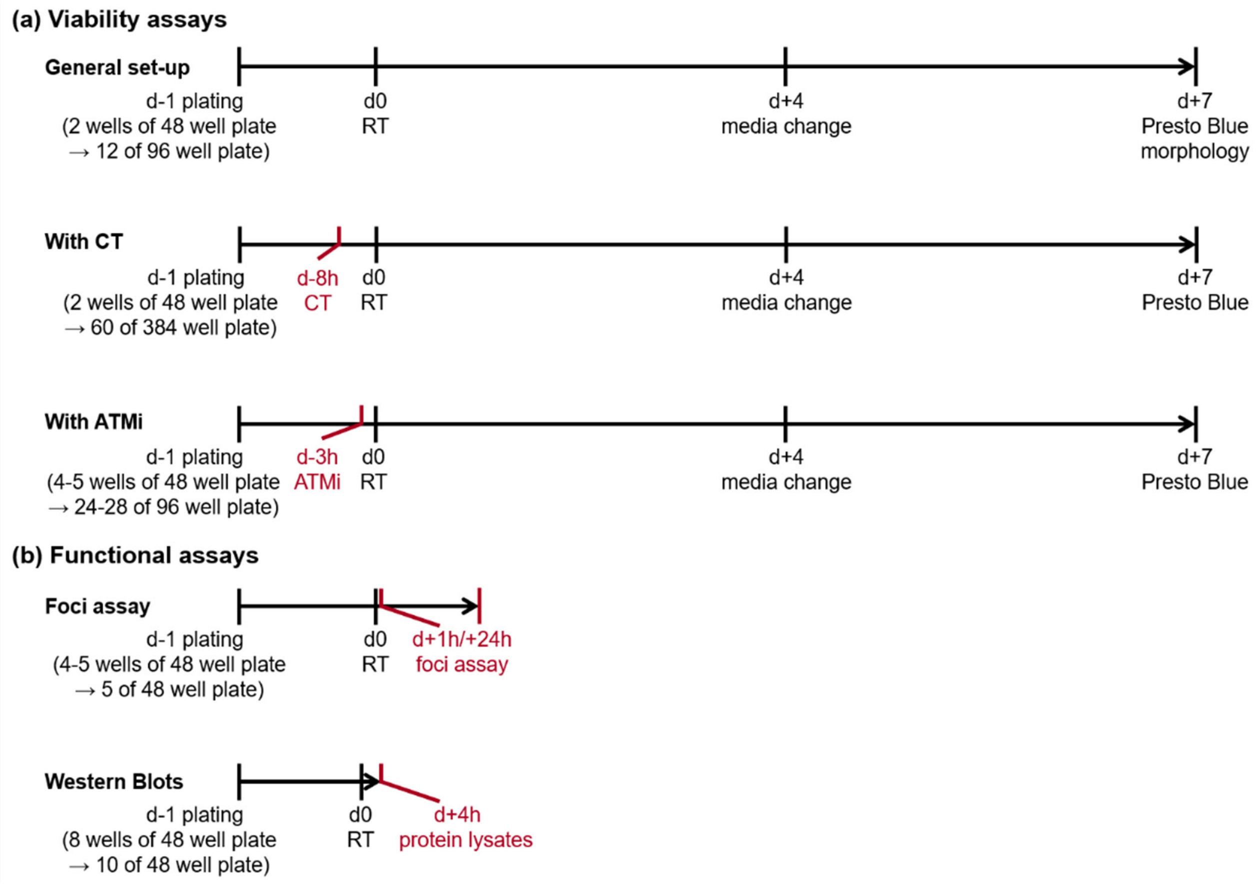

Figure S1. Timelines of experiments: **(a)** viability and **(b)** functional assays.

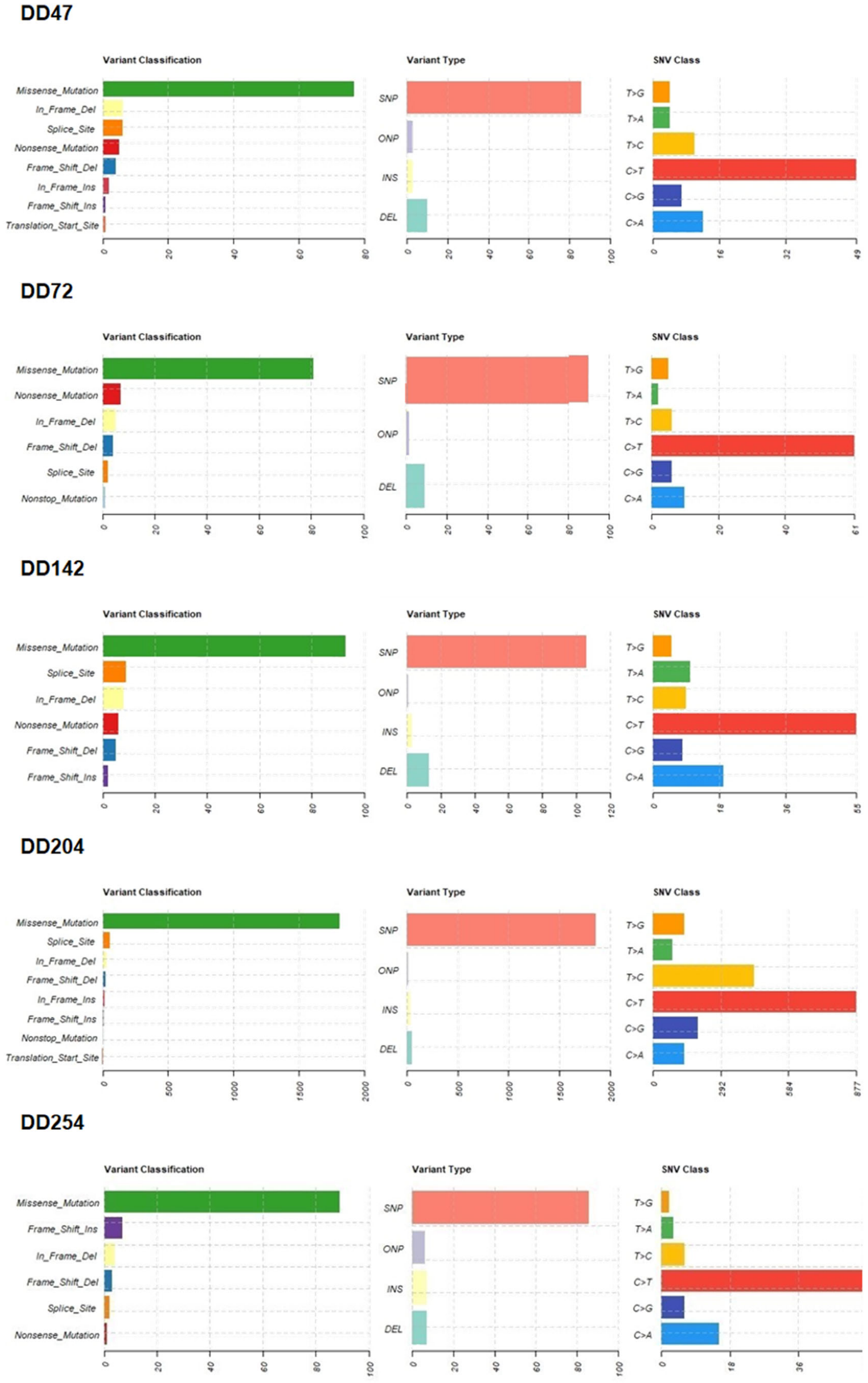

Figure S2: Variant classification of CRC PDOs.

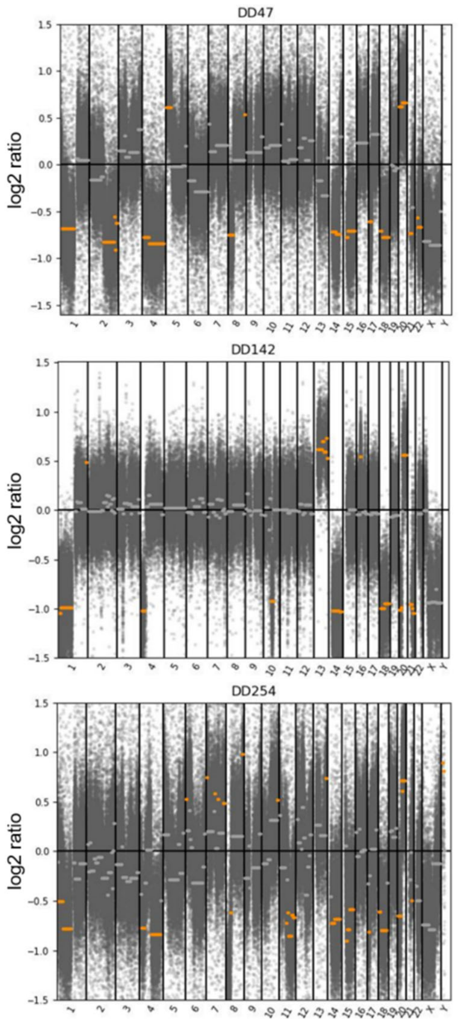

Figure S3: CNVs in CRC PDOs versus pooled normal organoids.

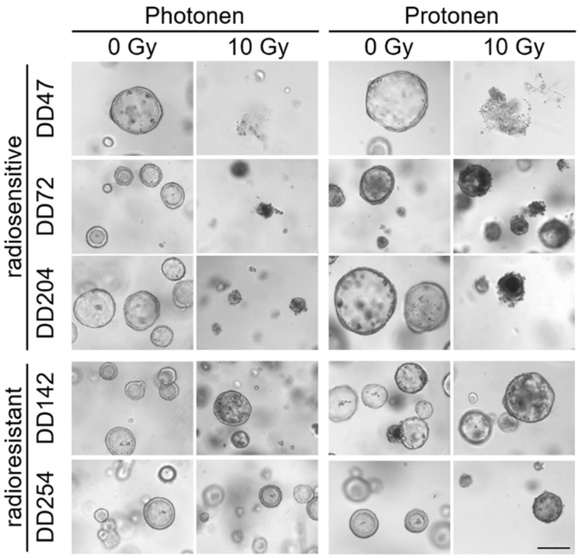

Figure S4: Exemplary pictures of organoid morphology upon control (0 Gy) and 10 Gy irradiation. Scale bar: 200 µM.

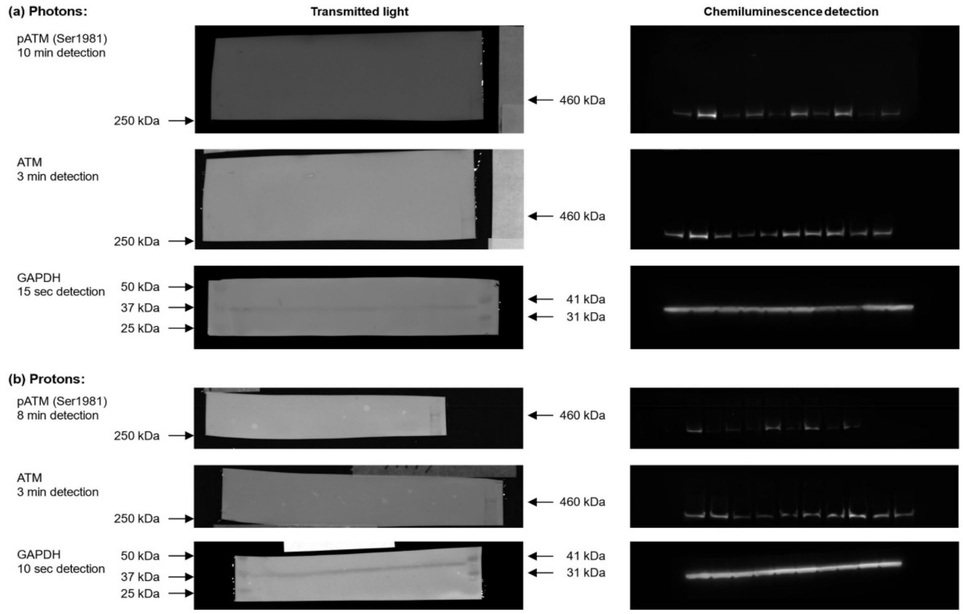

(c) Loading order of each blot: Marker Precision Plus Protein All Blue Standard
DD47 0 Gy
DD47 6 Gy
DD72 0 Gy
DD72 6 Gy
DD142 0 Gy
DD142 6 Gy
DD204 0 Gy
DD204 6 Gy
DD254 0 Gy
DD254 6 Gy
Marker HiMark Prestained HMW Protein Standard

(d) Quantification:

		Photons									
		DD47		DD72		DD142		DD204		DD254	
		0 Gy	6 Gy	0 Gy	6 Gy	0 Gy	6 Gy	0 Gy	6 Gy	0 Gy	6 Gy
pATM	Integrated Density minus background	3.644.314	15.957.005	2.777.779	6.147.874	2.461.174	9.429.076	3.604.769	11.028.412	2.426.802	4.901.121
	normalized to GAPDH	0,17	0,81	0,14	0,33	0,12	0,42	0,22	0,82	0,11	0,19
	**ratio of bands**	**1,00**	**4,75**	**0,83**	**1,91**	**0,69**	**2,43**	**1,30**	**4,78**	**0,63**	**1,12**
	**ratio to 0 Gy of each PDO**	**1,00**	**4,75**	**1,00**	**2,31**	**1,00**	**3,53**	**1,00**	**3,69**	**1,00**	**1,78**
ATM	Integrated Density minus background	6.669.897	13.563.379	5.283.378	2.699.967	3.767.245	8.536.508	7.516.649	8.764.663	5.313.553	7.331.760
	normalized to GAPDH	0,31	0,69	0,27	0,14	0,18	0,38	0,46	0,65	0,24	0,29
	**ratio of bands**	**1,00**	**2,20**	**0,86**	**0,46**	**0,58**	**1,20**	**1,48**	**2,08**	**0,76**	**0,92**
	**ratio to 0 Gy of each PDO**	**1,00**	**2,20**	**1,00**	**0,53**	**1,00**	**2,09**	**1,00**	**1,41**	**1,00**	**1,22**
GAPDH	Integrated Density minus background	21.339.499	19.690.489	19.635.852	18.849.385	20.898.479	22.709.324	16.273.195	13.500.968	22.500.946	25.545.613
	ratio of bands	1,00	0,92	0,92	0,88	0,98	1,06	0,76	0,63	1,05	1,20

		Protons									
		DD47		DD72		DD142		DD204		DD254	
		0 Gy	6 Gy	0 Gy	6 Gy	0 Gy	6 Gy	0 Gy	6 Gy	0 Gy	6 Gy
pATM	Integrated Density minus background	839.958	12.419.086	2.882.075	6.503.828	4.060.940	16.478.093	4.457.937	15.303.674	4.065.655	8.593.385
	normalized to GAPDH	0,03	0,50	0,12	0,27	0,15	0,62	0,20	0,70	0,16	0,32
	**ratio of bands**	**1,00**	**14,93**	**3,65**	**8,13**	**4,56**	**18,36**	**6,02**	**20,89**	**4,85**	**9,58**
	**ratio to 0 Gy of each PDO**	**1,00**	**14,93**	**1,00**	**2,23**	**1,00**	**4,03**	**1,00**	**3,47**	**1,00**	**1,98**
ATM	Integrated Density minus background	14.476.057	15.351.558	5.482.875	4.910.405	7.278.365	11.000.165	13.192.985	18.552.324	12.495.038	10.993.428
	normalized to GAPDH	0,58	0,62	0,23	0,21	0,27	0,41	0,60	0,85	0,50	0,41
	**ratio of bands**	**1,00**	**1,07**	**0,40**	**0,36**	**0,47**	**0,71**	**1,03**	**1,47**	**0,86**	**0,71**
	**ratio to 0 Gy of each PDO**	**1,00**	**1,07**	**1,00**	**0,88**	**1,00**	**1,50**	**1,00**	**1,42**	**1,00**	**0,82**
GAPDH	Integrated Density minus background	25.069.629	24.828.774	23.585.117	23.875.312	26.599.887	26.788.259	22.113.509	21.864.812	25.031.458	26.783.889
	ratio of bands	1,00	0,99	0,94	0,95	1,06	1,07	0,88	0,87	1,00	1,07

Figure S5. Original Western blots belonging to Figure 5. Western blot analysis of CRC PDOs 4 h post (a) photon and (b) proton irradiation for (phosphorylated) ATM. GAPDH served as a loading control. (c) The loading order of each blot is listed. (d) Quantification was performed with ImageJ.

Table S1. Patient's characteristics. The average growth rate of the PDOs was determined by Presto Blue assay.

Patient ID	Sex	Age [*1]	Diagnosis	TNM classification	Average growth rate [*2]	Neoadjuvant therapy	Tumor Regression [*3]
DD47	M	63	Recto-sigmoid cancer	pT2, pN0, M0	0.89	-	-
DD72	M	54	Rectal cancer	ypT3c, ypN1a, M0	0.74	∑50.4Gy, 2 cycles 5-FU, FOLFOX	unknown
DD142	M	58	Local recurrence of rectal cancer	rypT4b, rypNx, M0	0.98	Primary tumor: ∑25 Gy, FOLFOX, XELOX Local recurrence: ∑40.8 Gy, 5-FU	1
DD204	M	58	Rectal cancer	ypT2, ypN0, M0	0.93	∑ 50.4 Gy, 2 cycles 5-FU	2-3
DD254	M	72	Recto-sigmoid cancer	pT3b, pN0, M1	0.99	-	-

[*1] at diagnosis; [*2] of PDOs per day, calculated as factor per day via PrestoBlue; [*3] according to Dworak et al. 2007.

Table S4. Statistical interaction effects of ATM inhibitors and radiotherapy.

	**AZ32 (0/3 µM) + irradiation (0/6 Gy)**		**KU-55933 (0/10 µM) + irradiation (0/6 Gy)**	
	**photons**	**protons**	**photons**	**protons**
**DD47**	Synergy - 27.6% $P = 0.233$	Synergy - 16.2% $P = 0.404$	Synergy - 18.7% $P = 0.708$	Synergy - 14.7% $P = 0.59626$
**DD72**	Antagonism + 17.8% $P = 0.711$	Synergy - 38.8% $P = 0.085$	Synergy - 24.8% $P = 0.541$	Synergy - 42.5% $P = 0.114$
**DD204**	Synergy - 46.7% $P = 0.275$	Synergy - 39.8% $P =$ **0.020**	Synergy - 68.1% $P =$ **0.026**	Synergy - 60.9% $P =$ **0.022**
**DD142**	Synergy - 48.8% $P =$ **0.003**	Synergy - 46.8% $P =$ **0.013**	Synergy - 65.5% $P =$ **0.043**	Synergy - 58.2% $P =$ **0.006**
**DD254**	Synergy - 26.8% $P = 0.157$	Synergy - 27.2% $P = 0.2085$	Synergy - 52.0% $P = 0.094$	Synergy - 42. 8% $P = 0.078$
